# Reducing the Length of Hospitalization for Premature Infants Using a Quality Improvement Project

**DOI:** 10.3390/jcm15041650

**Published:** 2026-02-22

**Authors:** Ilan Segal, Hanna Shaferman, Evgenia Gurevich, Shmuel Zangen

**Affiliations:** 1 Neonatal Intensive Care Unit (NICU), Barzilai University Medical Center, Ashkelon 7830604, Israel; ilanse@bmc.gov.il; 2 Pediatric Department, Barzilai University Medical Center, Ashkelon 7830604, Israel; hashafeman@gmail.com (H.S.); gurevichjeny@gmail.com (E.G.); 3 Health Science Faculty, Ben Gurion University of Negev, Beer Sheva’ 8410501, Israel

**Keywords:** preterm infants, NICU, discharge planning, quality improvement, length of stay

## Abstract

**Background/Objectives:** Preterms often experience unnecessary delays in discharge. This quality improvement (QI) project aimed to reduce length of stay (LOS) in the neonatal intensive care unit (NICU). **Methods:** In this before-and-after intervention study on preterms (28 + 0–33 + 6 weeks gestation), we compared NICU outcomes (LOS, postmenstrual age (PMA) and weight at discharge, and readmission within 7 days) from pre-intervention (2013–2015) and post-intervention (2016–2017) periods. The intervention included discharge planning, standardized checklists, parental education, and team-based coordination. **Results:** We analyzed 377 infants. In post-interventional group, there was a reduction in LOS and PMA at discharge (31.45 ± 19.05 vs. 26.8 ± 15.8 days, *p* = 0.01 and 35.93 ± 1.84 vs. 35.43 ± 1.27 weeks, *p* < 0.01, respectively) without significant differences in discharge weight and readmission rates. **Conclusions:** QI interventions were significantly associated with shorter LOS for preterms. Standardized discharge procedures may improve outcomes and resource utilization in NICUs.

## 1. Introduction

Premature infants born at early gestational ages are expected to have prolonged hospitalizations in neonatal intensive care units (NICUs). The most significant factors determining the length of hospitalization are gestational age and birth weight [1]. The earlier the gestational age and lower the birth weight, the higher the likelihood of significant complications, such as respiratory distress syndrome, bronchopulmonary dysplasia, necrotizing enterocolitis, intraventricular hemorrhage, periventricular leukomalacia, retinopathy of prematurity, and sepsis [1].

The discharge process for premature infants is complex and performed daily at NICUs. Discharge timing is influenced primarily by the complications experienced by the infant. However, in the absence of significant complications, discharge depends on the infant’s physiological maturity and ability to function as a healthy baby [1]. Key criteria include the ability to feed orally while gaining sufficient weight [2,3,4], maintain normal body temperature without mechanical support [5,6], and be free of apnea episodes related to prematurity.

Traditionally, these processes are expected to mature at around 35 weeks’ gestational age and a weight of approximately 2000 g. Determining the exact timing for discharge is challenging for medical teams, and early discharge can result in readmissions and illness [7], while late discharge exposes the infant to nosocomial infections, prolonged separation from parents, increased hospitalization length, and higher healthcare costs [8].

The mandatory processes before the infant is discharged home include routine follow-up, screening tests, and treatments based on the infant’s age, as well as parental education on proper care for the premature infant [9,10,11].

Optimally, infants should be discharged on the day they reach functional maturity and when they can safely stay at home. It is the responsibility of the medical staff, nursing staff, and parents to ensure there are no delays caused by poor planning. Recent quality improvement studies have highlighted various aspects of the discharge process of a preterm baby in NICUs [12,13,14,15]. Quality improvement projects addressing the discharge process for preterm infants consistently recommend standardized, multidisciplinary discharge planning that begins early in the NICU stay and incorporates caregiver education, assessment of discharge readiness, and structured communication tools. Key aspects include early and ongoing caregiver education on infant care, feeding, medication administration, and recognition of warning signs, with opportunities for hands-on practice and targeted feedback. Standardized education materials and delivery systems are emphasized to maximize impact and ensure consistency. Multidisciplinary team involvement is important in the discharge process. Discharge planning should involve physicians, nurses, social workers, and, when available, discharge coordinators or navigators to address medical, psychosocial, and logistical needs. The assessment of discharge readiness is based on both technical (e.g., feeding and medication administration) and emotional readiness of caregivers and should be evaluated, often using surveys or structured assessments. Structured communication and follow-up in the discharge process was also emphasized. Written and oral communication tools are restructured to clarify plans for the day, the stay, and the transition to home. Post-discharge follow-up (e.g., phone calls) is used to identify and mitigate care-related failures [12,13,14,15]. These practices are designed to reduce errors, improve caregiver confidence, and optimize outcomes for preterm infants transitioning from NICU to home.

Premature infants born at 28 + 0 to 33 + 6 weeks of gestation are usually more stable than extreme preterms. Hence, for this group, length of stay (LOS) is primarily influenced by organizational limitations such as laboratory, imaging, and subspecialty consultation availability rather than health-related risks. Infants born at earlier gestational ages (24 + 0 to 27 + 6 weeks, classified as extreme preterm) face more significant complications, which have a greater impact on discharge timing than staff actions. Additionally, the number of such infants in the NICU is small and inconsistent over time, statistically behaving as outliers and distorting results. Late preterm infants (34 + 0 weeks and above) are hospitalized for shorter durations, making it difficult to evaluate the discharge process within this timeframe. Moreover, early discharge in this group has been linked to increased morbidity and readmissions [16]. Recent studies touched on different aspects of premature infant discharge from the NICU. Important topics such as parent preparedness, expectations, and knowledge have become pivotal in the discharge process [17]. QIs tried to improve the timeliness of discharge home, focusing on teaching parents the skills to safely provide the differential care required by children at home given their prematurity condition. This process is conducted in the neonatal unit gradually in function of the clinical evolution of the children and the adaptation of the parents to the situation, considering their individual, family, social, and cultural characteristics [12].

Observations in the NICU revealed that premature infants without significant complications were often discharged several days after reaching functional maturity. This led to the initiation of a quality improvement project aimed at discharging premature infants as soon as they have achieved functional maturity, without unnecessary delays.

### Project Rationale and Study Objective

Standardizing the discharge process by introducing new guidelines and refining existing procedures could shorten LOS for moderate premature infants without increasing readmission rates. We hypothesized that LOS could be shortened without an increased readmission rate.

## 2. Materials and Methods

This study was conducted as part of a quality improvement initiative at the NICU at Barzilai Medical Center. Our ward is level 3, with 20 beds, at a NICU situated in Ashqelon, Southern Israel. Patients’ referrals originate from both rural and urban populations from the hospital’s catchment area with about 500,000 people. This was a before-and-after intervention study. A comparative analysis was performed between two periods: January 2013 to December 2015 (pre-intervention) and January 2016 to December 2017 (post-intervention). The data were retrieved from electronic medical charts. The data regarding infants’ readmissions within a week from discharge were retrieved from emergency and pediatrics wards’ electronic medical charts.

## 3. Population

Since the aim of our QI initiative was to target organizational delays rather than medical instability, we chose a group of preterms that are stable and would benefit the most from the initiative.

### 3.1. Inclusion Criteria

Preterm babies born at a gestational age of 28 + 0 to 33 + 6 weeks during the 2-year project period comprised the study group and were compared with those born during the 3-year pre-project period.

### 3.2. Exclusion Criteria

Preterms with diagnoses that were observed to have a major impact on LOS, such as congenital malformations, severe bronchopulmonary dysplasia, and surgical necrotizing enterocolitis, were excluded.

Primary outcomes were discharge weight, postmenstrual age (PMA) at discharge, LOS, and readmission rate within 1 week of discharge. Secondary outcomes were common prematurity complications.

### 3.3. Intervention

Definitions:

Our NICU functional maturity criteria are:

1. Full oral feeding for at least 48 h.
2. Normal weight gain.
3. Maintaining normal body temperature without mechanical support.
4. Free of apnea episodes related to prematurity for at least 72 h.

To streamline and improve the discharge process, several areas for intervention were identified, and a dedicated checklist that included 13 items (Appendix A) was inserted into infants’ medical records.

Areas for improvement were as follows:

1. Laboratory Tests: Complete blood count and chemistry should have been up to date at least 2 weeks prior to discharge. Imaging: A head ultrasound at postmenstrual age of 35 weeks should be performed.
2. Medications: Caffeine discontinuation was confirmed. Prescription for the infant specific medication was written.
3. Consultation: Subpecialty and all other health professions in place were involved if necessary.
4. Parental Training: Specifically, a resuscitation course and basic baby care, education, and guidance was performed.
5. Paperwork: Community clinic and pediatrician updated via a temporary discharge note.

The discharge nurse was responsible for coordinating the process in a timely manner, ticking the items in the checklist, and specifying the date. She then had to notify the physician in order to complete whichever item that was missing. The importance of working with the dedicated checklist was emphasized weekly on rounds. After introducing the checklist to the nursing staff, the implementation was immediate. To address the above issues and prevent unnecessary delays, the intervention was initiated when the attending neonatologist assumed that the infant would be ready for discharge, within 7–10 days. To reinforce and prepare parents for discharge, we elaborated on the discharge process during weekly parents’ meetings—round table meetings that are held by a senior neonatologist and where parents are warmly asked to rise any general concerns or questions. This was expected to align expectations from both sides and facilitate discharge.

We emphasize that during the study, period discharge criteria have not been changed. We used physiological maturity criteria in alignment with AAP policy statement [1]. Indications for caffeine use were apnea, patient receiving positive pressure ventilation, or pre-extubation and remained identical throughout the study years.

A wash-out period, which is usually used in before-and-after QI projects to allow the staff to learn and accommodate themselves to new methods, was not employed, since we did not introduce a new or sophisticated item to the nurses’ or physicians’ daily work.

### 3.4. Statistical Analysis

Descriptive statistics were generated for all study variables, including frequencies for categorical variables (e.g., infant gender) and means with standard deviations for continuous variables (e.g., gestational age). Differences between pre- and post-intervention groups were analyzed using chi-square tests for categorical data and Mann–Whitney (U) test for continuous data, with medians and IQRs. Analyses were conducted using SPSS version 25, with statistical significance set at 5% (*p* < 0.05).

## 4. Results

During the study period, a total of 377 premature infants meeting the inclusion criteria were treated at the NICU. Table 1 compares the demographic and clinical characteristics of infants born before and after the intervention. The findings show that most demographic and clinical parameters were comparable between the two periods. There was no significant difference in gender (males 111 (53.11%) vs. 82 (51.25%), *p* = 0.72), gestational age (*p* = 0.24), percentage of multiple pregnancy (88 (41.71%) vs. 71 (42.77%), *p* = 0.83), and in-hospital mortality (1 (0.008%) vs. 1 (0.007%), *p* = 0.9) between pre- and post-interventional groups. There was also no significant difference in the number of preterms with prematurity-related medical conditions such as retinopathy of prematurity (2 (3%) vs. 0 (0%), *p* = 0.12), abnormal head ultrasound (10 (6.49%) vs. 5 (4.31%), *p* = 0.44), significant PDA (7 (3.4%) vs. 3 (2.04%), *p* = 0.43), respiratory distress syndrome (94 (44.76%) vs. 73 (43.98%), *p* = 0.88), necrotizing enterocolitis treated conservatively (2 (0.95%) vs. 4 (2.41%), *p* = 0.26), severe early onset sepsis (4 (1.9%) vs. 0 (0%), *p* = 0.07), and steroid treatment (2 (1.21%) vs. 9 (4.27%)) between pre- and post-intervention groups. However, significant differences were observed in specific parameters. A significantly higher number of infants experienced apnea episodes in pre-intervention period compared to post-intervention (52 (24.88%) vs. 22 (13.33%), *p* = 0.001). Infants born during the pre-intervention period had higher average birth weight compared to those born during the post-intervention period (1709 vs. 1638 g, *p* = 0.04). The number of infants treated with caffeine was higher in the pre-intervention period than in the post-intervention period (85 (40.86%) vs. 49 (29.52%), *p* = 0.03).

Table 2 presents the primary research outcomes, highlighting differences between the pre- and post-intervention periods. The average LOS was significantly shorter in the post-intervention period (27 ± 12 vs. 30.5 ± 15 days, *p* = 0.02). The average PMA at discharge was significantly lower (35.93 ± 1.84 vs. 35.43 ± 1.27 weeks, *p* < 0.01) in post-intervention group compared to pre-intervention group. There was no significant difference in discharge weight and the percentage of babies discharged in weight percentile ≤ 10 (2100 ± 500 vs. 2050 ± 300 g, *p* = 0.08; 64 (32.65%) vs. 49 (34%), *p* = 0.79, respectively) between post- and pre-interventional groups.

Two of 200 babies (1%) in the pre-interventional group were readmitted to the NICU, while there were no readmissions in the post-interventional group, but this difference was not statistically significant (*p* = 0.22).

## 5. Discussion

This quality improvement project successfully reduced the average hospitalization duration for premature infants by approximately 4.5 days. The earlier discharge was also reflected in younger PMA (by approximately 3.5 days). While earlier discharge may benefit families and resource utilization, current evidence does not fully address long-term neurodevelopmental or maternal outcomes. The shorter LOS and younger PMA were probably the result of a better coordinated discharge process. We have included stable preterms born at a gestational age of 28 + 0 to 33 + 6 weeks and excluded preterms with medical conditions and complications that could influence the LOS, such as congenital malformations, severe bronchopulmonary dysplasia, and surgical necrotizing enterocolitis.

The American Academy of Pediatrics (AAP) policy statement from 2008 emphasizes that premature infants can be discharged based on physiological maturity rather than indices of weight or age, provided the following conditions are met: active parental involvement: parents participate in the discharge process; community health services: community health professionals are available to manage the infant’s care; and follow-up plans: a structured growth and developmental follow-up plan is in place [1].

Studies from resource-limited settings, such as Colombia and India, demonstrated that premature infants with low discharge weights (1300–1400 g) can be discharged safely without increasing readmission rates [8,11]. They found that the lack of organization and the absence of a field leader hinder the discharge of medically stable preterm infants. In another study from Sweden, which included 21 neonatal intensive care units (NICUs) and examined the length of hospitalization and postmenstrual age at discharge, differences of up to 2 weeks were found in the average length of hospitalization for preterm infants born at 30–34 weeks of gestation. The infant’s morbidity explained only 13% of the variance. The authors hypothesized that factors such as parental involvement, breastfeeding, and the presence of a supportive community system might influence the length of hospitalization [16]. Recent QI studies highlighted various aspects of preterm discharge process. Kaemingk et al. focused on improving discharge hour [12], while McFarlane tried to improve the discharge process by reducing “last-minute” parent–nurse education on the day of discharge [13]. A multicenter and interdisciplinary expert panel reached a consensus on multiple potential best practices for complex discharge preparations from regional children’s hospital [14]. A systematic review of seven qualitative studies found that although a high percentage of parents felt prepared for discharge, some parents still transition home feeling both excited and nervous. Therefore, there is a poor understanding of how prepared parents are for the discharge [15]. In a more recent systematic review, Wu et al. emphasized parents’ need for psychological support, knowledge, and skills in preterm infant care, as well as post-discharge support, as an important item of preterm baby discharge planning [16]. In our study, we showed that simple interventions, such as checklist implementation and early discharge planning, all managed by an assigned topic champion, may effectively reduce hospitalization duration for “well” preterm infants. Since our intervention was introduced as a bundle, it is difficult to sort out with confidence what the weight of each component was. Moreover, none of the items in the checklist was completely new to the team, and we assume that the organization and coordination of the process best impacted the results. The external validity of our study may be questioned, since we used a “dedicated” nurse for this QI initiative. We think that a simple, easy-to-use, and reasonable checklist would be adopted by nurses in low- and high-income countries alike. The smaller the unit, the easier the implementation process. Effective checklist development requires multidisciplinary collaboration, including neonatologists, nurses, case managers, and, when possible, family representatives. Involving frontline staff ensures the checklist is practical and relevant to daily workflow. The checklist should be tailored to the specific needs of preterm infants, such as gestational age assessment, technical skill requirements (e.g., feeding and medication administration), and alignment with national or institutional guidelines. Iterative modification based on user feedback and local context is essential to maintain clinical utility and acceptability [17,18,19,20,21].

In our study, there was a significant difference in several baseline characteristics between pre- and post-interventional groups, such as birth weight, apnea, and caffeine use. These baseline differences between the groups may serve as an alternative explanation for the main study results. Regarding apnea of prematurity, infants in the pre-intervention group had more apnea and caffeine use. These may act as confounders. Although, a recent RCT by Carlo et al. compared extended caffeine use, continued after discharge home with standard caffeine use. They concluded that continuation of caffeine did not have an effect on LOS [22], and this suggests that the extent of caffeine use may have a minor effect on the results.

### 5.1. Limitations

Our study has several limitations. This was a retrospective study. The pre- and post-intervention groups differed in mean birth weight and rate of apnea and caffeine use, and these were not adjusted for.

A wash-out period was not employed in the project. This period would allow the staff to learn and accommodate themselves to new techniques and methods and should improve the project results. We may speculate that using such a wash-out period could improve the results even further.

### 5.2. Room for Further Improvement

There are other proven interventions that may further shorten LOS in the NICU and could be evaluated in future QI [before-and-after] studies.

### 5.3. Transition to Open Cribs

In our NICU, infants are transferred from incubators to cribs when they weigh at least 1700 g, are stable, and no longer require respiratory support or IV fluids. Studies have shown that transitioning infants at 1600 g is safe and can shorten hospital stay [5,23]. Adjusting this practice to focus on temperature stability rather than weight might further facilitate a timely discharge and reduce hospitalization length.

### 5.4. Oral Feeding

In the past, transitioning to oral feeding occurred when infants reached 34 weeks’ gestation and a weight of 1700 g. Studies have shown that early introduction of oral feeding, guided by infant cues and parental involvement, can accelerate the transition to full oral feeding and result in earlier discharge [24]. At this time, our NICU adopted a “cue-based feeding” approach, which may further shorten hospitalization duration in the future.

### 5.5. Parents’ Involvement

Studies have shown that parents’ perspective is significantly different than physicians’ and nurses’ [16,17,18]. In future QIs, we plan to involve parents, using focus groups to better understands their needs.

### 5.6. Economic and Clinical Implications

Prolonged hospitalization increases healthcare system resource utilization. Although this study did not include a cost–benefit analysis, the 4.5-day reduction in hospital stays likely results in significant economic savings, particularly when applied to large populations. Additionally, shorter hospital stays can strengthen parent–infant bonding and, in theory, reduce complications associated with extended hospitalizations, such as nosocomial infections.

## 6. Conclusions

In this before-and-after quality improvement project, there was a shorter LOS in preterm infants born at 28 + 0 to 33 + 6 weeks gestation after the intervention. Identifying additional areas for intervention and standardizing criteria for functional maturity could enhance outcomes for NICU patients. Further projects may optimize discharge process for premature infants.

## Figures and Tables

**Table 1:** Comparison of demographic and clinical data of infants born before and after the intervention.

Variable	2013–2015 (Pre-Intervention, *n* = 211)	2016–2017 (Post-Intervention, *n* = 166)	*p*-Value
Gender (Male)	111 (53.11%)	82 (51.25%)	0.72
Multiple Pregnancy	88 (41.71%)	71 (42.77%)	0.83
Gestational Age			0.24
28 + 0–29 + 6 weeks	34 (16.11%)	19 (11.45%)	
30 + 0–31 + 6 weeks	64 (30.33%)	45 (27.11%)	
32 + 0–33 + 6 weeks	113 (53.55%)	102 (61.45%)	
Birth Weight (grams)	1638.3 ± 439.75	1709.33 ± 377.11	0.04
In-Hospital Mortality	1 (0.008%)	1 (0.007%)	0.9
Apnea Episodes	52 (24.88%)	22 (13.33%)	<0.01
Abnormal Head Ultrasound	10 (6.49%)	5 (4.31%)	0.44
Retinopathy of Prematurity	2 (3%)	0 (0%)	0.12
* Significant PDA	7 (3.4%)	3 (2.04%)	0.43
Respiratory Distress Syndrome	94 (44.76%)	73 (43.98%)	0.88
** Necrotizing Enterocolitis	2 (0.95%)	4 (2.41%)	0.26
Severe Early Onset Sepsis	4 (1.9%)	0 (0%)	0.07
Caffeine Treatment	85 (40.86%)	49 (29.52%)	0.03
*** Steroid Treatment	2 (1.21%)	9 (4.27%)	0.14

* Significant PDA—according to pediatric cardiologist assessment ** Necrotizing enterocolotis, Bells stage II or greater—conservative treatment. *** Steroid treatment—we used the DART protocol for dexamethasone to wean from ventilator or from non-invasive ventilation.

**Table 2:** Comparison of discharge outcomes for infants born before and after the intervention.

Variable	2013–2015 (Pre-Intervention, *n* = 200)	2016–2017 (Post-Intervention, *n* = 156)	*p*-Value
Length of Stay (days), (mean ± SD)	30.5 ± 15	27 ± 12	0.02
Discharge Weight (grams), (mean ± SD)	2100 ± 500	2050 ± 300	0.08
Weight Percentile at Discharge, (mean ± SD)	30.50 ± 12	31.00 ± 22.00	0.81
Postmenstrual Age at Discharge (weeks), (mean ± SD)	35.93 ± 1.84	35.43 ± 1.27	<0.01
Percentage Discharged in Weight Percentile ≤10	64 (32.65%)	49 (34.03%)	0.79
Readmission Rate Within 1 Week	2 (1%)	0 (0%)	0.22

## Data Availability

Data are available on request.

## Abbreviations

NICU, neonatal intensive care unit; LOS, length of stay; IUGR, intrauterine growth restriction; QI, quality improvement; PMA, postmenstrual age.

## Appendix A

**Preterm Infant Discharge Checklist/Tickbox Date:**

**Laboratory:**

▯ Recent hematocrit/CBC taken? (should be no longer than 2 weeks)

**Imaging:**

▯ Was discharge US performed?

**Consultation:**

▯ ROP screening performed?
▯ Echocardiography performed?

**Other:**

▯ Audiology: AABR and OAE performed?
▯ Newborn screening test performed?

**Medications & immunization**

▯ Was caffeine discontinued at least 5 days prior to discharge?
▯ All necessary immunization given?

**Paperwork**

▯ Preliminary discharge letter given to family?
▯ Community pediatrician contacted?

**Nursing**

▯ Basic CPR training for parents?
▯ Baby care training?
▯ Parents ready for discharge?
